# Pathobiology and Treatment of Hepatitis Virus-Related Thrombocytopenia

**DOI:** 10.4084/MJHID.2009.023

**Published:** 2009-11-25

**Authors:** Roberto Stasi, Lian Wea Chia, Pallavi Kalkur, Robert Lowe, Muriel S. Shannon

**Affiliations:** Department of Haematology, St George’s Hospital, London, UK

## Abstract

Thrombocytopenia is a well recognized complication of infections, including those from hepatotropic viruses. Thrombocytopenia may actually be the only manifestation of vital hepatitis, which should therefore be considered in the differential diagnosis of primary immune thrombocytopenia (ITP). The mechanisms of thrombocytopenia associated with viral hepatitis vary widely depending on the specific infectious agent and the severity of liver disease. Most of the studies have described thrombocytopenia in association with chronic hepatitis C virus (HCV) infection, the most common cause of chronic infection worldwide. Studies have shown that treatment of HCV infection often results in substantial improvement or complete recovery of the thrombocytopenia. In patients with thrombocytopenia associated with HCV-related chronic liver disease, the use of eltrombopag, a thrombopoietin receptor agonist, normalizes platelet levels thereby permitting the initiation of antiviral therapy.

## Introduction:

Thrombocytopenia, either alone or in combination with other hematologic abnormalities, is commonly associated with infectious diseases^1^. However, only a few studies have specifically investigated this condition in patients with viral hepatitis^1^.

The onset of the thrombocytopenia is rarely abrupt and severe during acute viral hepatitis A (HAV)^2^–^5^, B (HBV)^5^–^7^, C (HCV)^6^ and E (HEV)^9^–^11^. This appears to have a course similar to that of the thrombocytopenia associated with self-limiting infections in children, such as varicella, rubella, or mumps^7^. It is, at least in part, mediated by immune complexes and generally resolves spontaneously within 2 to 8 weeks. In occasional individuals it may persist for months before remitting. Virus-associated hemophagocytic syndrome has been reported in several cases of HAV infection^8^–^11^, and rarely in association with HBV and HCV infections^12^–^14^.

On the other hand, persistent thrombo-cytopenia is more typically associated with chronic infection from HBV or HCV. Despite chronic HBV infection is still highly endemic in areas such as South East Asia and Africa^15^, there is a paucity of data about HBV and thrombocytopenia. In one study in treatment-naïve patients with chronic hepatitis B, thrombocytopenia (defined as a platelet counts below 150 x 10^9^/l) was observed in 17.7% of 219 patients with chronic active hepatitis B and 10.6% of 123 HBV inactive carriers^16^. Treatment of HBV infection with interferon-alpha (IFN-alpha) is also frequently complicated with thrombo-cytopenia, which in some series has been described in over 60% of cases^17^.

However, most of the recent literature pertaining to thrombocytopenia is about chronic hepatitis C, which will be the focus of our review.

## Natural History of HCV Infection:

HCV is now recognized as the most common viral infection causing chronic liver disease in humans. The 3rd National Health and Nutrition Examination Survey (NHANES III) estimated that nearly 3.2 million persons in the general population of the United States are infected with HCV^18^. Worldwide, an estimated 145 million individuals (2.2% of the world’s population) are infected^19^. HCV infection evolves towards a chronic state in approximately 85% of patients as demonstrated by the persistence of HCV-RNA in serum^20^. However, severe and long-term complications of chronic HCV infection such as liver cirrhosis, end-stage liver disease, and hepatocellular carcinoma develop only in a proportion of infected patients, after a period that can exceed 10 to 20 years^21^. Chronic HCV infection has also been reported to be associated with the development of several extrahepatic alterations, including thrombo-cytopenia^22^.

## Hcv - Associated Thrombocytopenia

### Epidemiology:

Table 1 summarizes the results on the prevalence of HCV infection from several cross-sectional studies in adult patients fulfilling the diagnostic criteria for immune thrombocytopenia (ITP) of the American Society of Hematology (ASH)^23^. Altogether, serologic evidence of HCV infection was found in 159 of 799 (20%) cases. The major series published to date evaluated 250 patients^23^. A positive serology was found in 76 (30%) of these patients.

While retrospective studies^24^,^25^ suggest that the prevalence of ITP among HCV patients is greater than would be expected by chance, the prevalence of HCV-positive ITP patients in some cohorts may be indirectly related to the background prevalence of HCV infection reported in the general populations^23^,^26^–^28^. Chiao et al calculated the incidence rate of ITP among 120,691 HCV-infected and 454,905 matched HCV-uninfected US veterans who received diagnoses during the period 1997 to 2004^29^. Their results indicate that HCV infection is actually associated with an elevated risk of developing ITP (HR, 1.8; 95% CI, 1.4–2.3) among both untreated and treated patients. Pockros et al retrospectively identified 7 ITP cases among 3440 new HCV patients seen over a 56-month period^24^. They estimated that the prevalence of CITP among their HCV patients was much greater than would be expected by chance (P < .00001). Even in the presence of active liver disease, patients with HCV infection present with lower platelet counts when compared with patients with HBV or alcoholic liver disease^23^,^25^. Thrombocytopenia either pre-exists and prevents the initiation of treatment with pegylated interferon (PEG-IFN) or develops as a consequence of PEG-IFN treatment, leading to dose modification in 19% of cases and discontinuation in 2% of cases^30^. In patients with cirrhosis, thrombocytopenia complicates antiviral treatment much more frequently than in patients with HCV infection without cirrhosis^31^.

### Pathophysiology:

A variety of pathogenic mechanisms are reported to be implicated in thrombocytopenia related to chronic HCV infection. These include: 1) sequestration of platelets in the enlarged spleen secondary to portal hypertension (hyper-splenism); 2) reduced hepatic production of thrombopoietin; 3) bone marrow suppression by HCV or antiviral treatment; and 4) increased platelet destruction mediated by immune mechanisms involving anti-platelet autoantibodies and platelet-associated immune complexes.

- 1) Hypersplenism is a common finding in patients with advanced liver disease (especially cirrhotic patients), who develop portal hypertension ultimately resulting in an enlarged spleen and subsequent platelet sequestration. The increased portal pressure causes redistribution of blood to the spleen, subsequent pooling of platelets, and the increased clearance of platelets from the circulation^32^. There is an inverse correlation between spleen size and platelet count in patients with chronic liver disease, the vast majority of whom have HCV infection^33^,^34^. However, splenic platelet pooling does not fully account for the occurrence of thrombocytopenia in chronic liver disease. In fact, approximately one third of patients with splenomegaly have normal platelet counts^34^, hypersplenism is not a consistent finding in patients with HCV-positive patients with thrombocytopenia^26^,^35^, spleen volume and platelet count have not been closely correlated in cirrhotic patients with end-stage liver disease and thrombocytopenia^36^, and thrombocytopenia has been shown to persist after surgical portal decompression in cirrhotic patients^37^,^38^.

- 2) Reduced hepatic production of the thrombopoietin (TPO) may be one of the contributing causes of thrombocytopenia during chronic liver diseases. TPO, the growth factor that primarily regulates megakaryocyte maturation and platelet formation, is produced mainly by hepatocytes, and is normally released at a constant rate into the circulation^39^. Circulating TPO binds to the TPO receptor (also referred to as c-Mpl) on hematopoietic stem cells and on megakaryocytes, and promotes all stages of platelet production, from early megakaryocyte proliferation to mega-karyocytic maturation and platelet formation. TPO also binds to platelets and enhances platelet activation and function. In turn, platelets not only bind TPO, but also internalize and degrade TPO. Thus, serum levels of TPO are normally regulated by the total platelet mass, including platelets sequestered in the spleen, rather than by the production rate of TPO^39^. Under normal conditions, if platelet production decreases, the circulating platelet count subsequently falls, less TPO is bound to platelets, and consequently the plasma TPO concentration increases. As a result, megakaryocytopoiesis increases to restore platelet homeostasis, resulting in more platelets produced and released. Once the platelet count increases, excess TPO is bound by circulating platelets, and TPO levels decrease to normal levels. However, in patients with extensive liver cirrhosis and or fibrosis and subsequent reduction in functioning hepatocytes (clinically manifested as severe impairment of liver function), production of TPO can be reduced^34^,^40^. Studies have demonstrated an inverse correlation between serum TPO levels and liver fibrosis grade (r = 0.50; P < 0.0001), with thrombocytopenia occurring with greater frequency and severity in patients with grade 3 or 4 liver fibrosis than in those with grades 0–2 liver fibrosis^34^,^40^. A correlation between low TPO serum level and decreases in global liver function (r = 0.52; P = 0.01) has also been demonstrated^40^. One study of thrombocytopenic patients with cirrhotic liver disease demonstrated a reduction in platelet production in the bone marrow in the presence of low blood TPO levels^41^. This finding suggests that the bone marrow of such patients may produce fewer platelets because of decreased TPO levels, resulting in thrombocytopenia. Furthermore, after successful orthotopic liver transplantation, when the dysfunctional liver is replaced by a functioning organ, plasma TPO levels increase, followed by increased platelet production and by an improvement in thrombocytopenia^36^,^42^. Increased degradation of TPO, mediated by the binding of TPO to platelets sequestered in the enlarged spleen, may also contribute to thrombocytopenia in patients with cirrhosis. Serum TPO levels and platelet counts can increase significantly after partial splenic embolization and the normal physiological relationship between TPO and platelet count has been restored following this procedure^43^. Serum TPO levels in patients with chronic liver disease do not reflect TPO production because of the complex interactions between TPO production, TPO degradation, platelet turnover and thrombocytopenia. In this patient population, serum TPO levels have been variously reported to be low, normal or elevated, in the presence of thrombocytopenia, without close correlation between TPO and platelet counts^43^,^44^. In contrast, patients with high grade (grades 3–4) liver fibrosis have significantly lower TPO levels than patients with less severe fibrosis (liver fibrosis grades 0–2), reflecting decreased production of the TPO by the damaged liver^34^.

- 3) Impaired production of platelets secondary to HCV infection is thought to be a contributing factor in the development of thrombocytopenia. In a study using reticulated platelets in peripheral blood as a marker of thrombopoiesis, patients with liver cirrhosis had low platelet production^41^. Also, the decrease in HCV viral load following interferon (IFN)-alpha treatment correlates with significant increases in platelet count^24^ in the absence of hypersplenism or serological evidence of platelet autoantibodies in some cases^26^. Finally, recent data on short-term CFU-MK assays in patients with chronic hepatitis C showed an evident depression in the number of colony-forming unit-megakaryocyte (CFU-meg). The whole of these data suggest that HCV can directly affect megakaryopoiesis.

However, antiviral therapy is itself associated with thrombocytopenia. IFN administration can induce a rapid and sustained reduction in peripheral platelet count. Peck-Radosavljevic and colleagues demonstrated that the platelet count decreased by nearly 28% in subjects treated with at least one dose of standard IFN and pegylated interferon (PEG)^45^. They also found that despite a corresponding increase in serum TPO levels, the reticulated platelet count did not change or actually decreased among subjects continuously exposed to PEG. These data indicate that bone marrow suppression, rather than increased platelet consumption, is the primary mechanism responsible for IFN-related thrombocytopenia. Interestingly, IFN-alpha treatment may also suppress the production or secretion of TPO^45^. Concomitant ribavirin therapy appears to have a protective effect against IFN-induced reductions in platelet count. In randomized controlled trials of PEG with or without ribavirin (RBV), the median decrease in platelet count was smaller among those receiving RBV therapy compared with those receiving only PEG^45^. Overall, in such trials, severe grade 4 thrombocytopenia (less than 20 x 10^9^/l) was not observed, and platelet counts less than 50 x 10^9^/l rarely were observed. Current guidelines therefore recommend the management of mild-to-moderate thrombocytopenia with PEG dose reduction and the discontinuation of HCV therapy in cases of severe thrombocytopenia, which typically is seen only in the setting of cirrhosis.

- 4) Although there is a higher prevalence of thrombocytopenia and anti-platelet antibodies in patients with liver disease caused by HCV than in patients with hepatitis B infection^25^, the pathogenic significance of anti-platelet antibodies is uncertain^46^. Using a direct monoclonal antibody-specific immobilization of platelet antigen assay (MAIPA), detectable platelet antibodies were found in 32 of 48 (66%) HCV-infected individuals at various stages of disease^46^. The most common target was glycoprotein IIb/IIIa, but all other glycoproteins were also targets. However, platelet autoantibodies lack specificity and do not assist in the diagnosis of immune thrombocytopenia. Recently, however, the pathogenetic role of platelet antibodies has been supported by an elegant study showing that HCV core envelope 1 can induce thrombocytopenia by molecular mimicry with an epitope on platelet surface integrin GPIIIa, GPIIIa49–66^47^.

Other studies have shown that HCV-RNA can be detected in washed platelets of infected individuals, particularly if thrombocytopenic^48^. Furthermore, there is a non-saturable binding of HCV to platelets^49^. High affinity binding of HCV to platelet membrane with subsequent binding of anti-HCV antibody could theoretically lead to “innocent bystander” phagocytosis of platelets^49^. The improvement of thrombocytopenia after successful interferon therapy supports this kind of mechanism.

### Clinical manifestations:

In one study from Japan^50^ the platelet counts in HCV-positive patients were lower than in the HCV-negative patients (26 ± 9 *vs* 49 ± 30 x 10^9^/l, respectively; P<0.02). Conversely, in an American study carried out in the Los Angeles area^23^, fewer HCV-positive patients had severe thrombocytopenia, defined as platelet count ≤10 x 10^9^/l (4% vs. 46% for ITP, P≤0.001). However, 56 (74%) patients had a platelet count ≤50 x 10^9^/l. Symptoms and signs of thrombocytopenia were less frequent in HCV-positive ITP, but major bleeding was more frequent (25% vs. 10%, P=0.0059). Serum cryoglobulins and anticardiolipin antibodies were more frequent in HCV-positive ITP (90% and 62% respectively), but rare in HCV-negative ITP (7% and 15%, P≤0.001 compared with HCV-positive ITP). In the French^51^ and Chinese^27^ studies the characteristics of ITP in HCV-positive patients did not differ from HCV-negative ones.

### Treatment:

Most case series of patients with HCV infection and chronic immune thrombocytopenia have reported a greater than 50% platelet response to steroids^23^,^24^,^52^,^53^. Only in the study from Sakuraya et al none of the 10 HCV-positive patients treated with prednisolone achieved a response^50^. Response to splenectomy was not found to differ significantly between HCV-positive and HCV-negative patients in two studies describing patients with chronic ITP^27^,^50^.

Rajan *et al* noted that only a minority of HCV-positive patients received some form of treatment for thrombocytopenia [29 (38%) vs. 158 (91%) for HCV-negative ITP]^23^. Of the seven patients treated with prednisone (4 responded, 57%), six developed elevations of hepatic transaminases of greater than twice pretreatment levels while receiving prednisone. All six patients had a documented increase in HCV viral load. Two patients developed elevated serum bilirubin levels, with one patient developing overt jaundice. Treatment with either intravenous immunoglobulin (IVIG) or anti-RhD Ig proved effective in increasing platelet counts in both the HCV seropositive and seronegative patients. Of five HCV-positive patients treated with interferon-alpha (IFN-α), four responded with increased platelet counts. Responders to IFN-α could be distinguished from the non-responder by a decrease in HCV quantitative RNA, hepatic transaminases and cryoglobulins^54^.

In the report of Garcia-Suarez et al each of 6 HCV patients treated with IFN-α responded with a significant increase in platelet count^26^. Iga et al reported significant increases in the platelet counts of 12 HCV infected patients who were complete responders to interferon alpha (IFN-α) treatment, but no improvement in the platelet counts of 11 patients who failed IFN-α therapy assessed by viral load^35^.

Considering the results of these various studies^26^,^35^,^54^, approximately half HCV-positive adult ITP patients treated with IFN-α responded with a rise in platelet count.

Research has focused on developing compounds specifically to stimulate thrombopoietin (TPO) activity in order to prevent or treat thrombocytopenia in chronic liver diseases. Eltrombopag is a small-molecule nonpeptide oral platelet growth factor that acts as an agonist to the thrombopoietin-receptor^55^. A phase II multi-center, randomized trial of daily eltrombopag in patients with HCV-associated thrombocytopenia and compensated liver disease showed that after 4 weeks of therapy platelet count increased to ≥100 x 10^9^/L in 75%, 79%, and 95% of patients treated with 30 mg, 50 mg, and 75 mg eltrombopag, respectively, compared to no response in placebo patients (P < 0.001) 56. Significantly more patients in the eltrombopag treatment groups (36%, 53%, and 65% in the 30-mg, 50-mg, and 75-mg groups) completed 12 weeks of antiviral therapy compared with 6% of placebo patients and 75% of these patients had platelet counts above baseline values at the end of the antiviral treatment phase. The most common adverse event during the initial 4 weeks was headache, reported in 36%, 16%, and 17% of patients who received 30 mg of eltrombopag, 50 mg of eltrombopag, and 75 mg of eltrombopag, respectively, as well as in 17% of patients who received placebo. Thereafter, the adverse events were those expected with interferon-based therapy (influenza-like illness, fatigue, chills, and headache).

Since eltrombopag has shown remarkable activity in chronic ITP as well^57^, this agent appears to be an adequate candidate for the management of HCV-related chronic thrombocytopenia.

## Conclusions:

Both acute and chronic viral hepatitis may be associated with isolated thrombocytopenia and should be considered in the differential diagnosis of ITP when isolated thrombocytopenia is present. Most of the studies have described thrombocytopenia in association with chronic HCV infection, by far the most common cause of chronic hepatitis. Several potential mechanisms can contribute to the thrombocytopenia in chronic HCV infection, including accelerated platelet clearance due to immune complex disease, cross-reactivity of anti-platelet glycoprotein antibodies and viral or bacterial antibodies, defective platelet production, and splenic sequestration of platelets secondary to portal hypertension and decreased production of thrombopoietin.

Serologic evaluation for HCV infection is indicated in patients with ITP because of the potential adverse effect of prolonged corticosteroid usage on the underlying infection and the utility of antiviral therapy in treating the both the underlying infection and the thrombocytopenia. Treatment with eltrombopag, a second generation thrombopoietin receptor agonist, appears to be very efficacious in elevating the platelet count and is well tolerated. Ongoing phase III studies in HCV-related chronic thrombocytopenia will better define the benefits of this agent compared to standard of care.

## Figures and Tables

**Table 1.** Prevalence of HCV infection in adult ITP patients.

Authors	Total number	Number of infected (%)
Pawlotsly et al. (1995)^51^	139*	14 (10)
Pivetti et al. (1996)^28^	33	12 (36)
Garcia-Suarez et al (2000)^26^	51	13 (22)
Sakuraya et al (2002)^50^	79	11 (14)
Zhang et al. (2003)^27^	247	33 (13)
Rajan et al (2005)^23^	250	76 (30)
Total	799	159 (20)

* Seven patients of this series had an associated autoimmune disorder. Study only included patients with platelet counts of less than 25x10^9^/L
